# Bioproduction and extraction of jasmonates

**DOI:** 10.1186/1753-6561-8-S4-P201

**Published:** 2014-10-01

**Authors:** Laís Baichi Buttarello, Alexandre Zanelli dos Santos, Murilo Daniel de Mello Innocentini, Miriam Verginia Lourenço

**Affiliations:** 1 Chemical Engineering Course, UNAERP, University of Ribeirao Preto, SP, Brazil; 2 Biotechnology Unit, UNAERP, University of Ribeirao Preto, SP, Brazil

## Introduction

Jasmonates are a growing class of compounds of vegetable origin which present phytoregulator activity and toxicity against some types of cancer cells. Additionally the jasmonates have also shown activity against some types of nematodes [1]. Jasmonates available on the market are of plant origin or synthetic, present costs are still high. Like plants, some microorganisms have the potential to produce jasmonates. Some microorganisms have the potential to produce this class of compounds, including filamentous fungus Botryosphaeria rhodina has shown to be the most promising. The choice of the strain and optimization of process steps are fundamental premises for increasing the scale of production [2]. Furthermore, the optimization of the steps of extraction and purification of the product in the fermentation broth are essential to ensure the viability of the process. Currently, studies of recovery of jasmonates produced by Botryosphaeria rhodina are based on organic solvents. In this work, an alternative recovery route is proposed, based on adsorption, and the determination of the adsorption kinetics of jasmonates present in fermented using ion exchange resins Amberlite ^® ^is presented.

## Methodology

Fermentations were conducted in Erlenmeyer of 250 mL using 50 mL of culture medium M2 and 5 mL of inoculum. After a period of fermentation, the mycelium was removed by vacuum filtration and subjected to fermentation extraction tests. To do this the filtrate collected was adjusted to pH 3.0. Then it was mixed in an Erlenmeyer of 100 mL of fermented and 1 g of resins Amberlite ^® ^XAD-2, XAD-4 or XAD-7 separately, put under stirring at 130 rpm for 60 minutes [3]. Samples (5 mL) were collected after 2, 4, 6, 8, 10, 20, 40 and 60 minutes of contact and subsequently subjected to extraction in order to determine the content of jasmonates remaining in each sample. The extractions were performed by liquid-liquid partition using ethyl acetate as solvent extractor. The monitoring was performed by CCDC (Thin Layer Chromatography Comparative) using silica chromatoplates with UV indicator. The quantification of jasmonates was performed by HPLC (High Performance Liquid Chromatography) using a chromatograph SHIMADZU (LC-10AD VP) coupled to a detector of diode array. It was used a m) and a solvent systemμSupelcosil C18 column (25 cm × 4.6 mm id, 5 comprised of MeOH:acetic acid 0.1% (60:40). The solvent flow was 0.85 mL.min-1 with analysis monitored at 210 nm. For quantification it was used the external standard method, by plotting a calibration curve with standard solutions of pure AJ at concentrations ranging from 0.1 - 1.0 mg.mL-1.

## Results and conclusions

The results showed that the efficiency of XAD-2 resin was 84.02%, XAD-4 was 96.3% and XAD-7 95.15%. With the data analyzed, it was concluded that even though the Amberlite XAD-4 has a slightly higher efficiency in the time of equilibrium, the resin XAD-7 after 40 minutes showed higher adsorption (93.01%) over XAD-4 (85.19%). Therefore, for the continuation of studies, the Amberlite ^® ^XAD-7 will be used.
